# 4-(4-Methoxy­phen­yl)-1-phenyl­pyridine-2,6(1*H*,3*H*)-dione

**DOI:** 10.1107/S1600536809017942

**Published:** 2009-05-20

**Authors:** Ushati Das, Shardul B. Chheda, Suhas R. Pednekar, Narendra P. Karambelkar, T. N. Guru Row

**Affiliations:** a Solid State and Structural Chemistry Unit, Indian Institute of Science, Bangalore 560 012, India; b F-12, Organic Chemistry Research Laboratory, Ramanujan Ruia College, Matunga (East), Mumbai 400 019, India; c Jai Research Foundation, C-12, Road No. 16, Wagale Industrial Estate, Thane (West) 400 064, India

## Abstract

In the title compound, C_18_H_15_NO_3_, the pyridine-2,6-dione ring adopts an envelope conformation. The phenyl ring lies approximately perpendicular to the mean plane of the pyridine-2,6-dione ring [dihedral angle = 81.5 (1)°], while the methoxy­phenyl ring is tilted to the same plane by a dihedral angle of 34.8 (1)°. Inter­molecular C—H⋯O inter­actions link the mol­ecules into chains along [100].

## Related literature

For background literature concerning pyridine-2,6-diones, see: Kon & Nanji (1933 ▶). For ring conformation analysis, see: Cremer & Pople (1975 ▶).
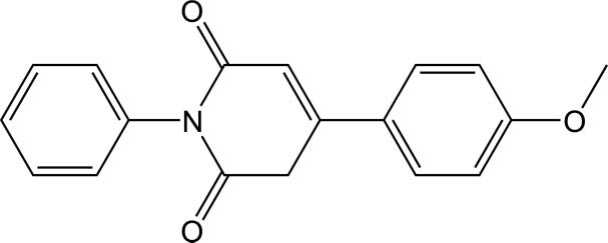


## Experimental

### 

#### Crystal data

C_18_H_15_NO_3_
*M* *_r_* = 293.31
Triclinic, 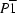

*a* = 7.4652 (5) Å
*b* = 9.0885 (7) Å
*c* = 11.1181 (8) Å
α = 77.384 (1)°
β = 88.747 (2)°
γ = 77.493 (1)°
*V* = 718.40 (9) Å^3^
*Z* = 2
Mo *K*α radiation
μ = 0.09 mm^−1^
*T* = 292 K
0.52 × 0.41 × 0.32 mm

#### Data collection

Bruker SMART APEX CCD diffractometer
Absorption correction: multi-scan (*SADABS*; Sheldrick, 1996 ▶) *T*_min_ = 0.921, *T*_max_ = 0.970
7298 measured reflections
2714 independent reflections
2233 reflections with *I* > 2σ(*I*)
*R*_int_ = 0.017

#### Refinement

*R*[*F* ^2^ > 2σ(*F* ^2^)] = 0.040
*wR*(*F* ^2^) = 0.105
*S* = 0.91
2714 reflections
200 parameters
H-atom parameters constrained
Δρ_max_ = 0.15 e Å^−3^
Δρ_min_ = −0.17 e Å^−3^

### 

Data collection: *SMART* (Bruker, 2004 ▶); cell refinement: *SAINT* (Bruker, 2004 ▶); data reduction: *SAINT*; program(s) used to solve structure: *SHELXS97* (Sheldrick, 2008 ▶); program(s) used to refine structure: *SHELXL97* (Sheldrick, 2008 ▶); molecular graphics: *ORTEP-3 for Windows* (Farrugia, 1997 ▶) and *CAMERON* (Watkin *et al.*, 1993 ▶); software used to prepare material for publication: *PLATON* (Spek, 2009 ▶).

## Supplementary Material

Crystal structure: contains datablocks global, I. DOI: 10.1107/S1600536809017942/bi2366sup1.cif

Structure factors: contains datablocks I. DOI: 10.1107/S1600536809017942/bi2366Isup2.hkl

Additional supplementary materials:  crystallographic information; 3D view; checkCIF report

## Figures and Tables

**Table 1:** Hydrogen-bond geometry (Å, °)

*D*—H⋯*A*	*D*—H	H⋯*A*	*D*⋯*A*	*D*—H⋯*A*
C4—H4⋯O2^i^	0.93	2.52	3.4260 (18)	164.00
C17—H17*B*⋯O1^ii^	0.96	2.55	3.073 (2)	114.00

Symmetry codes: (i) 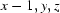; (ii) 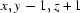.

**Fig. 1.**
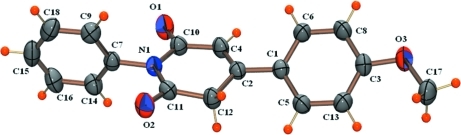
Molecular structure with displacement ellipsoids drawn at the 50% probability level. H atoms are shown as small spheres of arbitrary radius.

**Fig. 2.**
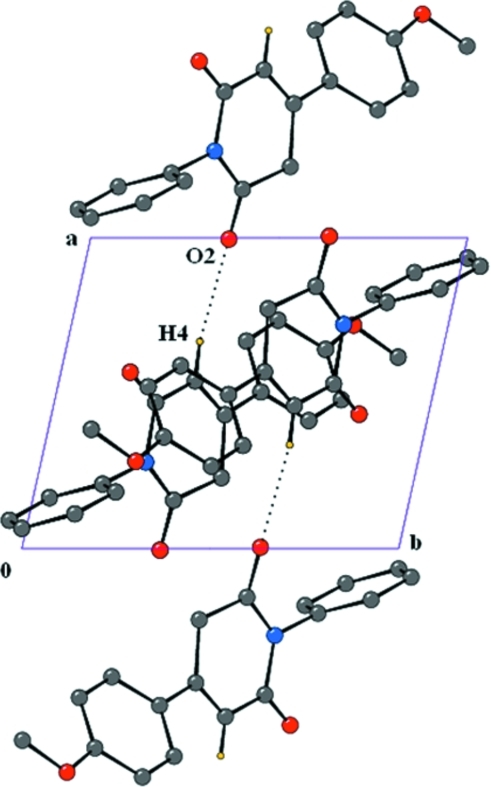
Packing diagram viewed down the c axis. The dotted lines indicate intermolecular C—H···O interactions. H atoms not involved in these interactions are omitted.

**Fig. 3.**
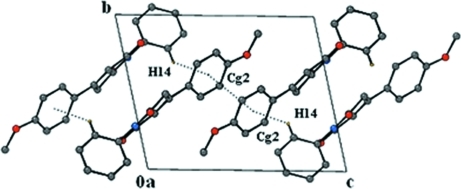
Packing diagram viewed down the a axis. The dotted lines indicate intermolecular C—H···π and π···π interactions. H atoms not involved in these interactions are omitted.

## supplementary crystallographic information

### Comment

The title compound belongs to the pyridine-2,6-dione family (Kon & Nanji, 1933). Pyridine-2,6-diones have a π-conjugated system and an extremely reactive methylene group, which has been exploited to carry out various condensations to form fused ring systems. The Mannich reaction is one of the important processes in medicinal chemistry that introduces an aminomethyl group, which acts as a pharmacophore when introduced into an appropriate substrate, and the title compound has been exploited *via* this reaction to form a wide array of compounds.

In the crystal structure, the pyridine-2,6-dione ring [N1/C10/C4/C2/C12/C11] adopts an envelope conformation (Cremer & Pople, 1975) with puckering parameters *q*_2_ = 0.1394 (15)°, *q*_3_ = 0.0581 (15)°, *φ* = 247.1 (6)°, puckering amplitude Q = 0.1510 (16) Å. The phenyl ring is nearly perpendicular to the pyridine-2,6-dione ring (torsion angle C11—N1—C7—C9 = 100.49 °) while the methoxy phenyl ring is tilted to the plane of the heterocyclic ring with a torsion angle C4—C2—C1—C6 = 31.14 °. Within the aryl rings, the C—C bonds are in the range of 1.343 (3) to 1.381 (3) Å, which is in accordance with those found in similar structures. The C—N single bonds (1.388 (3) to 1.448 (3) Å), C—O single bonds (1.365 (3) to 1.426 (3) Å), and C=O double bonds (1.213 (3) to 1.220 (2) Å) are within the usual range. The structure contains intermolecular C—H···O hydrogen bonds (see Table and Figure 2) which link the molecules into chains along the *a* axis. Alternating C—H···π and π···π interactions are also present (Figure 3).

### Experimental

The title compound was prepared by microwave treatment of 3-(4-methoxyphenyl)-2-pentene-1,5-dioic acid with acetic anhydride to form the corresponding dione, which was then reacted further with aniline to give the resultant compound (yield 96%). ^1^H NMR (DMSO): δ 6.98 (*m*, 9H), 3.87 (*s*, 3H), 3.97(*s*, 2H), 6.67 (*s*, 1H).

### Refinement

H atoms were placed geometrically with C—H = 0.93–0.97 Å and refined as riding with *U*_iso_(H) = 1.2 or 1.5 *U*_eq_(C).

### Crystal data

**Table:**

C_18_H_15_NO_3_	*Z* = 2
*M**_r_* = 293.31	*F*(000) = 308
Triclinic, *P*1	*D*_x_ = 1.356 Mg m^−^^3^
Hall symbol: -P 1	Mo *K*α radiation, λ = 0.71073 Å
*a* = 7.4652 (5) Å	Cell parameters from 2604 reflections
*b* = 9.0885 (7) Å	θ = 2.4–28.0°
*c* = 11.1181 (8) Å	µ = 0.09 mm^−^^1^
α = 77.384 (1)°	*T* = 292 K
β = 88.747 (2)°	Block, colourless
γ = 77.493 (1)°	0.52 × 0.41 × 0.32 mm
*V* = 718.40 (9) Å^3^	

### Data collection

**Table:**

Bruker SMART APEX CCD diffractometer	2714 independent reflections
Radiation source: fine-focus sealed tube	2233 reflections with *I* > 2σ(*I*)
graphite	*R*_int_ = 0.017
φ and ω scans	θ_max_ = 25.7°, θ_min_ = 1.9°
Absorption correction: multi-scan (*SADABS*; Sheldrick, 1996)	*h* = −9→9
*T*_min_ = 0.921, *T*_max_ = 0.970	*k* = −11→11
7298 measured reflections	*l* = −13→13

### Refinement

**Table:**

Refinement on *F*^2^	Primary atom site location: structure-invariant direct methods
Least-squares matrix: full	Secondary atom site location: difference Fourier map
*R*[*F*^2^ > 2σ(*F*^2^)] = 0.040	Hydrogen site location: inferred from neighbouring sites
*wR*(*F*^2^) = 0.105	H-atom parameters constrained
*S* = 0.91	*w* = 1/[σ^2^(*F*_o_^2^) + (0.0537*P*)^2^ + 0.2183*P*] where *P* = (*F*_o_^2^ + 2*F*_c_^2^)/3
2714 reflections	(Δ/σ)_max_ < 0.001
200 parameters	Δρ_max_ = 0.15 e Å^−^^3^
0 restraints	Δρ_min_ = −0.17 e Å^−^^3^

### Special details

**Table:**

Geometry. Bond distances, angles *etc*. have been calculated using the rounded fractional coordinates. All su's are estimated from the variances of the (full) variance-covariance matrix. The cell e.s.d.'s are taken into account in the estimation of distances, angles and torsion angles
Refinement. Refinement of *F*^2^ against ALL reflections. The weighted *R*-factor *wR* and goodness of fit *S* are based on *F*^2^, conventional *R*-factors *R* are based on *F*, with *F* set to zero for negative *F*^2^. The threshold expression of *F*^2^ > σ(*F*^2^) is used only for calculating *R*-factors(gt) *etc*. and is not relevant to the choice of reflections for refinement. *R*-factors based on *F*^2^ are statistically about twice as large as those based on *F*, and *R*- factors based on ALL data will be even larger.

### Fractional atomic coordinates and isotropic or equivalent isotropic displacement parameters (Å^2^)

**Table:**

	*x*	*y*	*z*	*U*_iso_*/*U*_eq_	
O1	0.43237 (14)	0.81573 (14)	−0.09890 (10)	0.0584 (4)	
O2	1.00465 (13)	0.63224 (13)	0.07261 (10)	0.0538 (4)	
O3	0.28108 (15)	0.25107 (13)	0.62184 (10)	0.0569 (4)	
N1	0.72144 (15)	0.72212 (13)	−0.01738 (10)	0.0388 (4)	
C1	0.49575 (18)	0.45771 (15)	0.29553 (12)	0.0369 (4)	
C2	0.56872 (18)	0.54010 (15)	0.18235 (12)	0.0370 (4)	
C3	0.3591 (2)	0.31101 (17)	0.51532 (13)	0.0418 (5)	
C4	0.46008 (19)	0.63892 (17)	0.09213 (13)	0.0420 (5)	
C5	0.5880 (2)	0.31397 (17)	0.36111 (13)	0.0428 (5)	
C6	0.33201 (19)	0.52602 (17)	0.34314 (13)	0.0432 (5)	
C7	0.79229 (18)	0.82360 (16)	−0.11708 (13)	0.0387 (4)	
C8	0.2655 (2)	0.45429 (18)	0.45097 (14)	0.0465 (5)	
C9	0.8359 (2)	0.95658 (17)	−0.09782 (14)	0.0462 (5)	
C10	0.53018 (19)	0.73075 (16)	−0.01525 (13)	0.0406 (4)	
C11	0.84395 (19)	0.62585 (16)	0.07338 (13)	0.0397 (5)	
C12	0.77132 (19)	0.51251 (18)	0.16928 (13)	0.0446 (5)	
C13	0.5210 (2)	0.24011 (17)	0.46966 (13)	0.0449 (5)	
C14	0.8175 (2)	0.78459 (18)	−0.23010 (14)	0.0475 (5)	
C15	0.9349 (2)	1.0118 (2)	−0.30544 (16)	0.0576 (6)	
C16	0.8880 (2)	0.8799 (2)	−0.32436 (15)	0.0567 (6)	
C17	0.3837 (3)	0.1142 (2)	0.70023 (15)	0.0585 (6)	
C18	0.9086 (2)	1.04971 (19)	−0.19256 (16)	0.0541 (6)	
H4	0.33372	0.64938	0.09840	0.0504*	
H5	0.69755	0.26599	0.33143	0.0513*	
H6	0.26674	0.62196	0.30098	0.0518*	
H8	0.15638	0.50240	0.48115	0.0558*	
H9	0.81648	0.98333	−0.02173	0.0554*	
H12A	0.82601	0.50985	0.24829	0.0536*	
H12B	0.81319	0.41097	0.15146	0.0536*	
H13	0.58454	0.14339	0.51153	0.0539*	
H14	0.78734	0.69496	−0.24275	0.0570*	
H15	0.98420	1.07481	−0.36893	0.0692*	
H16	0.90396	0.85501	−0.40126	0.0680*	
H17A	0.50078	0.13092	0.72080	0.0878*	
H17B	0.31768	0.08907	0.77430	0.0878*	
H17C	0.40147	0.03050	0.65833	0.0878*	
H18	0.94004	1.13880	−0.17976	0.0650*	

### Atomic displacement parameters (Å^2^)

**Table:**

	*U*^11^	*U*^22^	*U*^33^	*U*^12^	*U*^13^	*U*^23^
O1	0.0395 (6)	0.0727 (8)	0.0496 (6)	−0.0081 (5)	−0.0031 (5)	0.0114 (6)
O2	0.0335 (6)	0.0649 (7)	0.0596 (7)	−0.0153 (5)	−0.0013 (5)	−0.0016 (5)
O3	0.0537 (7)	0.0605 (7)	0.0484 (6)	−0.0121 (5)	0.0116 (5)	0.0042 (5)
N1	0.0332 (6)	0.0435 (7)	0.0380 (6)	−0.0104 (5)	0.0028 (5)	−0.0035 (5)
C1	0.0359 (7)	0.0391 (7)	0.0369 (7)	−0.0111 (6)	0.0015 (6)	−0.0081 (6)
C2	0.0363 (7)	0.0381 (7)	0.0382 (7)	−0.0104 (6)	0.0043 (6)	−0.0100 (6)
C3	0.0423 (8)	0.0474 (8)	0.0372 (8)	−0.0171 (7)	0.0044 (6)	−0.0056 (6)
C4	0.0311 (7)	0.0512 (9)	0.0434 (8)	−0.0120 (6)	0.0038 (6)	−0.0071 (7)
C5	0.0380 (8)	0.0453 (8)	0.0425 (8)	−0.0055 (6)	0.0057 (6)	−0.0083 (6)
C6	0.0403 (8)	0.0391 (8)	0.0454 (8)	−0.0057 (6)	0.0038 (6)	−0.0022 (6)
C7	0.0315 (7)	0.0421 (8)	0.0401 (8)	−0.0086 (6)	0.0009 (6)	−0.0035 (6)
C8	0.0379 (8)	0.0510 (9)	0.0481 (9)	−0.0074 (7)	0.0092 (7)	−0.0085 (7)
C9	0.0458 (9)	0.0458 (8)	0.0470 (9)	−0.0102 (7)	−0.0020 (7)	−0.0096 (7)
C10	0.0351 (7)	0.0454 (8)	0.0393 (8)	−0.0074 (6)	0.0007 (6)	−0.0061 (6)
C11	0.0345 (8)	0.0450 (8)	0.0401 (8)	−0.0086 (6)	0.0033 (6)	−0.0104 (6)
C12	0.0362 (8)	0.0514 (9)	0.0414 (8)	−0.0072 (6)	0.0013 (6)	−0.0021 (7)
C13	0.0465 (8)	0.0407 (8)	0.0434 (8)	−0.0067 (7)	−0.0006 (7)	−0.0029 (6)
C14	0.0451 (9)	0.0538 (9)	0.0471 (9)	−0.0167 (7)	0.0060 (7)	−0.0130 (7)
C15	0.0453 (9)	0.0647 (11)	0.0555 (10)	−0.0196 (8)	0.0034 (7)	0.0099 (8)
C16	0.0498 (10)	0.0783 (12)	0.0418 (9)	−0.0183 (9)	0.0090 (7)	−0.0094 (8)
C17	0.0640 (11)	0.0593 (10)	0.0460 (9)	−0.0164 (9)	0.0044 (8)	0.0046 (8)
C18	0.0501 (9)	0.0446 (9)	0.0655 (11)	−0.0177 (7)	−0.0054 (8)	0.0006 (8)

### Geometric parameters (Å, °)

**Table:**

O1—C10	1.2109 (18)	C11—C12	1.493 (2)
O2—C11	1.2133 (18)	C14—C16	1.379 (2)
O3—C3	1.3653 (18)	C15—C16	1.378 (2)
O3—C17	1.427 (2)	C15—C18	1.372 (2)
N1—C7	1.4485 (18)	C4—H4	0.930
N1—C10	1.4129 (19)	C5—H5	0.930
N1—C11	1.3886 (18)	C6—H6	0.930
C1—C2	1.4751 (19)	C8—H8	0.930
C1—C5	1.389 (2)	C9—H9	0.930
C1—C6	1.395 (2)	C12—H12A	0.970
C2—C4	1.341 (2)	C12—H12B	0.970
C2—C12	1.488 (2)	C13—H13	0.930
C3—C8	1.384 (2)	C14—H14	0.930
C3—C13	1.383 (2)	C15—H15	0.930
C4—C10	1.457 (2)	C16—H16	0.930
C5—C13	1.387 (2)	C17—H17A	0.960
C6—C8	1.372 (2)	C17—H17B	0.960
C7—C9	1.378 (2)	C17—H17C	0.960
C7—C14	1.377 (2)	C18—H18	0.930
C9—C18	1.380 (2)		
			
O1···C14	3.1810 (19)	C17···H13	2.5500
O1···C17^i^	3.073 (2)	C18···H17A^i^	3.1000
O1···C5^ii^	3.3926 (19)	C18···H6^iv^	2.9800
O2···C9	3.1501 (19)	H4···O2^x^	2.5200
O2···O2^iii^	3.1878 (16)	H4···C6	2.7100
O2···C11^iii^	3.1383 (19)	H4···H6	2.2700
O1···H9^iv^	2.8400	H5···C12	2.7000
O1···H17B^i^	2.5500	H5···H12A	2.5800
O2···H4^v^	2.5200	H5···H12B	2.4000
O2···H12B^iii^	2.8700	H5···C16^iii^	3.1000
O2···H18^vi^	2.7200	H6···C4	2.7000
O3···H12A^vii^	2.8300	H6···H4	2.2700
C2···C10^ii^	3.592 (2)	H6···C18^iv^	2.9800
C3···C6^vii^	3.556 (2)	H6···H18^iv^	2.5100
C4···C4^ii^	3.533 (2)	H8···H8^xi^	2.3700
C5···O1^ii^	3.3926 (19)	H9···O1^iv^	2.8400
C6···C3^vii^	3.556 (2)	H12A···C5	2.8600
C9···O2	3.1501 (19)	H12A···H5	2.5800
C10···C2^ii^	3.592 (2)	H12A···O3^vii^	2.8300
C11···C11^iii^	3.533 (2)	H12B···C5	2.9300
C11···O2^iii^	3.1383 (19)	H12B···H5	2.4000
C12···C14^iii^	3.588 (2)	H12B···O2^iii^	2.8700
C14···O1	3.1810 (19)	H12B···C14^iii^	2.9700
C14···C12^iii^	3.588 (2)	H13···C17	2.5500
C17···O1^viii^	3.073 (2)	H13···H17A	2.3800
C1···H14^ii^	2.9000	H13···H17C	2.3200
C4···H6	2.7000	H14···C1^ii^	2.9000
C5···H12A	2.8600	H14···C6^ii^	2.8100
C5···H12B	2.9300	H14···C8^ii^	3.0000
C6···H4	2.7100	H17A···C13	2.7600
C6···H14^ii^	2.8100	H17A···C18^viii^	3.1000
C8···H14^ii^	3.0000	H17A···H13	2.3800
C12···H5	2.7000	H17B···O1^viii^	2.5500
C13···H17C^ix^	3.0500	H17C···C13	2.7800
C13···H17C	2.7800	H17C···H13	2.3200
C13···H17A	2.7600	H17C···C13^ix^	3.0500
C14···H12B^iii^	2.9700	H18···O2^vi^	2.7200
C16···H5^iii^	3.1000	H18···H6^iv^	2.5100
			
C3—O3—C17	117.96 (13)	C9—C18—C15	120.48 (16)
C7—N1—C10	118.12 (11)	C2—C4—H4	118.00
C7—N1—C11	118.34 (11)	C10—C4—H4	118.00
C10—N1—C11	123.50 (12)	C1—C5—H5	119.00
C2—C1—C5	122.31 (13)	C13—C5—H5	119.00
C2—C1—C6	120.23 (12)	C1—C6—H6	119.00
C5—C1—C6	117.42 (13)	C8—C6—H6	119.00
C1—C2—C4	122.65 (13)	C3—C8—H8	120.00
C1—C2—C12	118.18 (12)	C6—C8—H8	120.00
C4—C2—C12	119.17 (13)	C7—C9—H9	120.00
O3—C3—C8	115.66 (13)	C18—C9—H9	120.00
O3—C3—C13	125.04 (14)	C2—C12—H12A	108.00
C8—C3—C13	119.30 (14)	C2—C12—H12B	108.00
C2—C4—C10	123.24 (13)	C11—C12—H12A	108.00
C1—C5—C13	121.73 (14)	C11—C12—H12B	108.00
C1—C6—C8	121.22 (14)	H12A—C12—H12B	107.00
N1—C7—C9	120.00 (13)	C3—C13—H13	120.00
N1—C7—C14	119.30 (13)	C5—C13—H13	120.00
C9—C7—C14	120.69 (14)	C7—C14—H14	120.00
C3—C8—C6	120.67 (14)	C16—C14—H14	120.00
C7—C9—C18	119.36 (14)	C16—C15—H15	120.00
O1—C10—N1	119.66 (13)	C18—C15—H15	120.00
O1—C10—C4	123.20 (13)	C14—C16—H16	120.00
N1—C10—C4	117.10 (12)	C15—C16—H16	120.00
O2—C11—N1	120.80 (13)	O3—C17—H17A	109.00
O2—C11—C12	121.64 (13)	O3—C17—H17B	109.00
N1—C11—C12	117.54 (12)	O3—C17—H17C	109.00
C2—C12—C11	117.14 (13)	H17A—C17—H17B	109.00
C3—C13—C5	119.65 (14)	H17A—C17—H17C	110.00
C7—C14—C16	119.27 (15)	H17B—C17—H17C	109.00
C16—C15—C18	119.70 (16)	C9—C18—H18	120.00
C14—C16—C15	120.49 (15)	C15—C18—H18	120.00
			
C17—O3—C3—C8	−171.64 (14)	C1—C2—C4—C10	174.69 (13)
C17—O3—C3—C13	8.0 (2)	C12—C2—C4—C10	−4.8 (2)
C10—N1—C7—C9	100.50 (16)	C1—C2—C12—C11	−163.73 (12)
C10—N1—C7—C14	−80.33 (17)	C4—C2—C12—C11	15.8 (2)
C11—N1—C7—C9	−77.12 (17)	O3—C3—C8—C6	179.52 (14)
C11—N1—C7—C14	102.05 (16)	C13—C3—C8—C6	−0.1 (2)
C7—N1—C10—O1	3.7 (2)	O3—C3—C13—C5	−178.98 (14)
C7—N1—C10—C4	−174.06 (12)	C8—C3—C13—C5	0.6 (2)
C11—N1—C10—O1	−178.78 (14)	C2—C4—C10—O1	177.20 (15)
C11—N1—C10—C4	3.4 (2)	C2—C4—C10—N1	−5.1 (2)
C7—N1—C11—O2	3.7 (2)	C1—C5—C13—C3	−0.6 (2)
C7—N1—C11—C12	−174.81 (12)	C1—C6—C8—C3	−0.4 (2)
C10—N1—C11—O2	−173.76 (13)	N1—C7—C9—C18	178.01 (13)
C10—N1—C11—C12	7.7 (2)	C14—C7—C9—C18	−1.2 (2)
C5—C1—C2—C4	151.27 (15)	N1—C7—C14—C16	−178.87 (13)
C5—C1—C2—C12	−29.3 (2)	C9—C7—C14—C16	0.3 (2)
C6—C1—C2—C4	−31.1 (2)	C7—C9—C18—C15	0.9 (2)
C6—C1—C2—C12	148.33 (14)	O2—C11—C12—C2	164.44 (14)
C2—C1—C5—C13	177.76 (14)	N1—C11—C12—C2	−17.04 (19)
C6—C1—C5—C13	0.1 (2)	C7—C14—C16—C15	0.8 (2)
C2—C1—C6—C8	−177.30 (14)	C18—C15—C16—C14	−1.0 (2)
C5—C1—C6—C8	0.4 (2)	C16—C15—C18—C9	0.1 (2)

Symmetry codes: (i) *x*, *y*+1, *z*−1; (ii) −*x*+1, −*y*+1, −*z*; (iii) −*x*+2, −*y*+1, −*z*; (iv) −*x*+1, −*y*+2, −*z*; (v) *x*+1, *y*, *z*; (vi) −*x*+2, −*y*+2, −*z*; (vii) −*x*+1, −*y*+1, −*z*+1; (viii) *x*, *y*−1, *z*+1; (ix) −*x*+1, −*y*, −*z*+1; (x) *x*−1, *y*, *z*; (xi) −*x*, −*y*+1, −*z*+1.

### Hydrogen-bond geometry (Å, °)

**Table:**

*D*—H···*A*	*D*—H	H···*A*	*D*···*A*	*D*—H···*A*
C4—H4···O2^x^	0.93	2.52	3.4260 (18)	164.00
C17—H17B···O1^viii^	0.96	2.55	3.073 (2)	114.00

Symmetry codes: (x) *x*−1, *y*, *z*; (viii) *x*, *y*−1, *z*+1.
